# 0986. The effect of eritoran on the severity of lung injury in two different rat lung injury models

**DOI:** 10.1186/2197-425X-2-S1-P71

**Published:** 2014-09-26

**Authors:** A Kundakci, H Verdi, E Ozturan Ozer, O Ozen Isiksacan, FB Atac, S Turkoglu, A Pirat

**Affiliations:** Baskent University Medical School, Ankara, Turkey

Toll like receptors (TLR) play an important role in noninfectious problems in critically ill patients. The central role of TLR4 in sepsis has been clearly demonstrated. However, the role of TLR as a therapeutic target in acute respiratory distress syndrome (ARDS) has not been studied.

The goal of this study is to compare the effects of eritoran, a TLR4 antagonist, on severity of ARDS in two different (pulmonary versus extrapulmonary) ARDS rat models.

Forty rats were randomized to sham (n=8), extrapulmonary control (n=8), extrapulmonary eritoran (n=8), pulmonary control (n=8), and pulmonary eritoran (n=8) groups. Intestinal ischemia-reperfusion (I/R) via ligation of superior mesenteric artery was used to induce the extrapulmonary ARDS model. Intratracheal instillation of hydrochloric acid was used for the pulmonary ARDS model. Rats received either eritoran 100 µg/kg or an equal volume of placebo solution. Serum tumor necrosis factor- α (TNF-α), interleukin-1β (IL-1β), and IL-6; lung tissue malondialdehyde (MDA), glutathione (GSH), and myeloperoxidase (MPO) levels; lung histopathologic examination; arterial partial oxygen pressure (pO_2_); and lung wet-to-dry weight ratio (w/d) measurements were used to compare and evaluate the severity of lung injury between the groups. TLR-4 and nuclear factor-κB (NF-κB) gene expressions were determined from lung tissue using polymerase chain reaction technique.

Compared to its control group, extrapulmonary eritoran group exhibited significantly less severe lung injury, as indicated by lower mean values for MDA (p< 0.05), MPO (p< 0.01), lung histopathologic injury score (p< 0.05), lung w/d weight ratio (p< 0.01), and higher mean values for pO_2_ (p< 0.01) and GSH (p< 0.05). Likewise mean values for serum TNF-α, IL-1β, and IL-6 levels were significantly lower than the control values in extrapulmonary eritoran group (p< 0.05 for all). TLR-4 and NF-κB gene expressions were significantly more pronounced in extrapulmonary control group than extrapulmonary eritoran group (p< 0.05 for both). Pulmonary control and eritoran groups were not significantly different in terms of mean values for MDA, GSH, and pO2 measurements (p>0.05 for all). However, mean values for MPO (p< 0.05), w/d weight ratio (p< 0.05), and lung histopathologic injury score (p< 0.05) were significantly higher in pulmonary control group than pulmonary eritoran group. Mean values for serum TNF-α, IL-1β, and IL-6 levels were not significantly different between pulmonary control and eritoran groups. The expression of NF-κB gene was not significantly different between the pulmonary control and pulmonary eritoran groups (p>0.05). However, compared to pulmonary control group the expression of TLR-4 gene was significantly less pronounced in the pulmonary eritoran group (p< 0.05).

In conclusion eritoran reduced the severity of lung injury both in pulmonary and extrapulmonary ARDS models. This effect of eritoran was more pronounced in the I/R lung injury model.

